# Long-lifetime water-washable ceramic catalyst filter for air purification

**DOI:** 10.1038/s41467-023-36050-w

**Published:** 2023-02-15

**Authors:** Hyuk Jae Kwon, Dong Sik Yang, Min Seok Koo, Sang Min Ji, Joonseon Jeong, Sehyeong Oh, Su Keun Kuk, Hyeon-su Heo, Dong Jin Ham, Mijong Kim, Hyoungwoo Choi, Jong-Min Lee, Joong-Won Shur, Woo-Jin Lee, Chang-Ook Bin, Nikolay Timofeev, Huiqing Wu, Liming Wang, Taewoo Lee, Daniel J. Jacob, Hyun Chul Lee

**Affiliations:** 1 Air Science Research Center (ASRC), Samsung Advanced Institute of Technology (SAIT), Samsung Electronics Co., Ltd., 130 Samsung-ro, Yeongtong-gu, Suwon-si, Gyeonggi-do 16678 Republic of Korea; 2 Corning Technology Center Korea, Corning Precision Material Co., Ltd., 212 Tangjeong-ro, Tangjeong-myeun, Asan-si, Chungcheongnam-do 31454 Republic of Korea; 3 Corning Scientific Center; 26, lit.A, Shatelena St., St. Petersburg 194021 Russia; 4 Corning Research Center China; Block H, 200 Jinsu Road, Shanghai, 201206 China; 5 Heesung Catalysts Co.; #507-1Da, 91, Somanggongwon-ro, Siheung-si, Gyeonggi-do 15088 Republic of Korea; 6 John A. Paulson School of Engineering and Applied Sciences, Harvard University, Cambridge, MA 02138 USA

**Keywords:** Chemical engineering, Pollution remediation, Photocatalysis

## Abstract

Particulate matter (PM) and volatile organic compounds (VOCs) are recognised as hazardous air pollutants threatening human health. Disposable filters are generally used for air purification despite frequent replacement and waste generation problems. However, the development of a novel regenerable and robust filter for long-term use is a huge challenge. Here, we report on a new class of facile water-washing regenerable ceramic catalyst filters (CCFs), developed to simultaneously remove PM (>95%) and VOCs (>82%) in single-pass and maximized space efficiency by coating the inner and outer filter channels with an inorganic membrane and a Cu_2_O/TiO_2_ photocatalyst, respectively. The CCFs reveal four-fold increase in the maximum dust loading capacity (approximately 20 g/L) in relation to conventional filters (5 g/L), and can be reused after ten regeneration capability with simple water washing retaining initial PM and VOC removal performances. Thus, the CCFs can be well-suited for indoor and outdoor air purification for 20 years, which shows a huge increase in lifetime compared to the 6-month lifespan of conventional filters. Finally, we believe that the development and implementation of CCFs for air purification can open new avenues for sustainable technology through renewability and zero-waste generation.

## Abstract (web summary)

Disposable filters are generally used for air purification despite frequent replacement and waste generation problems. Here, a ceramic catalyst filter is introduced as a new class of filter that simultaneously removes PMs and VOCs as primary air pollutants and can be regenerated and used for long periods by simple water washing.

## Introduction

Particulate matter (PM) and volatile organic compounds (VOCs) are hazardous air pollutants in outdoor and indoor air. The impact of these pollutants on human mortality is well-documented, in which PM is a leading cause of death^1^, and many VOCs are known carcinogens^2^. The general approach to controlling air pollution has been to decrease emissions. Air pollution from anthropogenic emissions is not just a local and acute problem; it is also a global and chronic problem as evidenced through terrible events such as the Great smog in London in 1952 caused by sulphates from coal fuel combustion and the Los Angeles smog in 1943 caused by nitrates from automotive emission. However, the removal of pollutants through air filtration can also be an effective approach both indoors and outdoors^3,4^.

Currently, the high-efficiency particulate air (HEPA) polymers and fibre filtration that utilises electrostatic force is considered to be the best solution for the removal of PMs^5,6^. However, the replacement and discarding of filters pose a serious issue leading to additional waste and generation of carbon emission from the incineration of used filters. To overcome this disadvantage, ceramic filters (CFs) used in automotive applications are considered as a suitable candidate for the next-generation filter supporting facile regeneration. CFs have generally been used to remove PM from the exhaust gases emitted from diesel engines, which involves two types of filtration mechanisms, namely deep-bed and cake filtrations. The dominant mechanism in a clean filter is initially deep-bed filtration during which the particulates are deposited inside the porous wall. As the dust load increases, a particulate layer (i.e., the “cake”) is formed along the wall surface in the inlet channels and cake filtration becomes the prevailing mechanism^7^. Soot, which is a PM, is initially captured through the deep-bed filtration and later deposited as a cake. However, the capture of PM via cake filtration would be more preferable because PM captured by deep-bed filtration cannot be easily removed from the CF during regeneration, which requires hot airflow and plasma flame at high temperatures^7,8^. As to the removal of VOC, activated carbons have generally been used with a sorption method under ambient conditions. However, they have similar problems to PM filters, which are disposable, have a low adsorption capacity, and cannot be used for long periods. To address these problems, a catalytic honeycomb CFs have been utilised. However, the automotive catalysts coated on the CF have been shown to be easily sintered and degraded during regeneration with high temperature^9–11^. Therefore, it is necessary to develop a filter that can be easily regenerated and simultaneously removes PM and VOC operated under ambient conditions. Thus, we considered photocatalysts as one of the most promising candidates owing to their unique properties such as strong oxidation ability, biological and chemical inactivity, and operation at room temperature^12^. UV photocatalytic oxidation (PCO) reactions using reactive oxygen species (ROS), such as hydroxyl radical (∙OH) and superoxide anion (∙O_2_^−^) generated from the photocatalyst under UV light irradiation, not only effectively remove and decompose VOCs^13,14^ but also disinfect bacteria and viruses^15^. Although their photocatalytic capability is limited, and their reaction rate is low due to their wide band–gap energy and fast recombination of the photo–induced electron–hole pairs^16,17^, they still have a great advantage of operating under ambient conditions such as room temperature, unlike general thermal catalysts.

Herein, we introduce a ceramic catalyst filter (CCF) as a new class of filter that simultaneously removes PMs and VOCs as primary air pollutants, and can be regenerated and used for long periods by simple water washing. Our concept aims to achieve a renewable robust filter wherein PMs are collected with the CF, VOC gases are decomposed by a coated photocatalyst operating under ambient conditions, and reuse is by simple washing with water. For proof of concept (PoC), we improved the PM removal efficiency by applying an additional inorganic membrane coating on the channel of the bare-CF for rapidly enhancing the cake filtration step. We also attempted to employ a UV-activated photocatalyst to decompose VOCs, which was coated over the outlet channel surface of the CF to prevent catalyst deactivation by PM. The enhanced photocatalytic activity was obtained by applying the developed Cu_2_O/TiO_2_ catalyst. In addition, we proved facile regeneration and use for long-lifetime of CCF compared with conventional HEPA filter by simple water-washing. Finally, we confirmed the CCF performance by employing a CCF filter panel system for purifying the in-flowing air in buildings and a free-standing air purification system as a proto located at the public spaces with ingress of outside air.

## Results

### Rational design concept of CCF

**Fig. 1:**
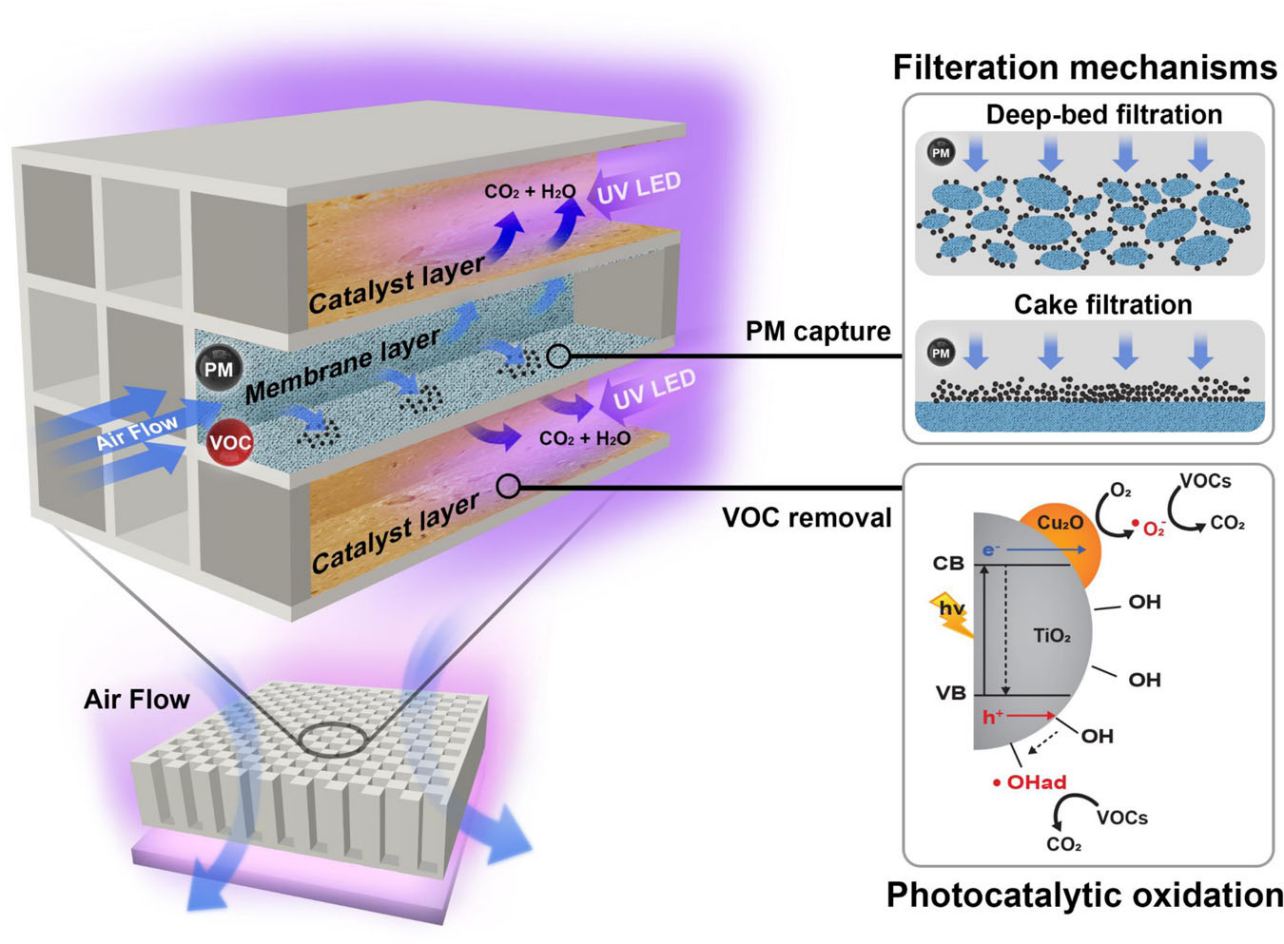
Design concept of Ceramic Catalyst Filter (CCF) system. The illustration shows a rational design concept for CCF preparation. Two main mechanisms, namely wall-flow filtration of PM by porous ceramic filter with two types of filtration mechanism, namely deep-bed and cake filtration, and photocatalytic perfect oxidation reaction for VOC removal by the developed Cu_2_O/TiO_2_ catalyst were applied to simultaneously remove the PM and VOC.

The design of CCF is based on a novel concept, wherein PMs are collected in the inlet channel of the ceramic filter, and VOC gases are decomposed by a photocatalyst coated in the outlet channel, under ambient conditions. This design concept of CCF can provide a definite advantage of being able to remove various air pollutants in the space of itself. The CF possesses an inlet channel with plugs and a porous inner wall, through which the air flows toward the outlet channel after penetrating the wall (Fig. 1). First, we selected a commercial CF designed with a conjugated plug to allow wall-flow through the porous wall for in-flowing air^18,19^. The filtration mechanism of CF to remove PM is a combination of deep-bed and cake filtrations, as shown in Fig. 1^7^. The greatest advantage of CF as PM removal filter is that the surface area over volume of the filter porous wall (m^2^/m^3^) is much larger than that of other filters prepared by polymers and fibres, which enables it to be used for longer durations^19^. Concerning regeneration, ceramics fired at high temperatures exhibit strong heat- and water-resistant properties and are also used as water-treatment membranes^20^. In addition to PM removal, we used it as a support for photocatalysts to oxidise VOCs to CO_2_ under ambient conditions without thermal sources, as shown in Fig. 1. The photocatalysts can remove VOC by the PCO reaction using ROS such as hydroxyl radical (∙OH) and superoxide anion (∙O_2_^−^) generated from the photocatalyst under UV light irradiation at room temperature. Our novel design concept entails that, along sequential air flow, PM are first captured by the ceramic porous wall at the inlet channel with initial deep-bed and following cake filtration mechanism, and then VOCs exiting the porous wall are decomposed using the PCO reaction by the UV–photocatalyst system at the outlet channel. Finally, to realise this concept, we also attempted an uncommon method of catalyst coating over the outlet channel surface for preventing catalyst deactivation by PM.

For the rational design of CCF, we prepared commercial ceramic filters with 100, 200, and 300 CPSI, where CPSI implies cells per square inch of the filter. To determine the optimal length of CCF, we investigated the pressure drop of the filters along the CF length using a pressure drop model developed by Masoudi et al^7^. and Konstandopoulos & Johnson^21^ (Supplementary Fig. 1). The flow characteristics such as distributions of velocity and pressure of CFs inside each CF channel were investigated using computational fluid dynamics (CFD) simulations (Supplementary Fig. 2a). Using this developed model, the porous-wall lengths were optimised to 60–120 mm, which indicate the ranges of minimum pressure drop (Supplementary Fig. 2b). The predicted pressure drop was estimated to be 71 Pa at an optimal porous-wall length of 105 mm (Supplementary Table 1). Based on these results, commercial CFs with a filter length of 115 mm with each plug of 5 mm were procured from Corning Co. with 200 CPSI and 8.0 mil wall thickness (0.203 mm). Thus, the CF having an optimal length was carefully employed to prepare the CCF.

### PM removal by surface treatment

**Fig. 2:**
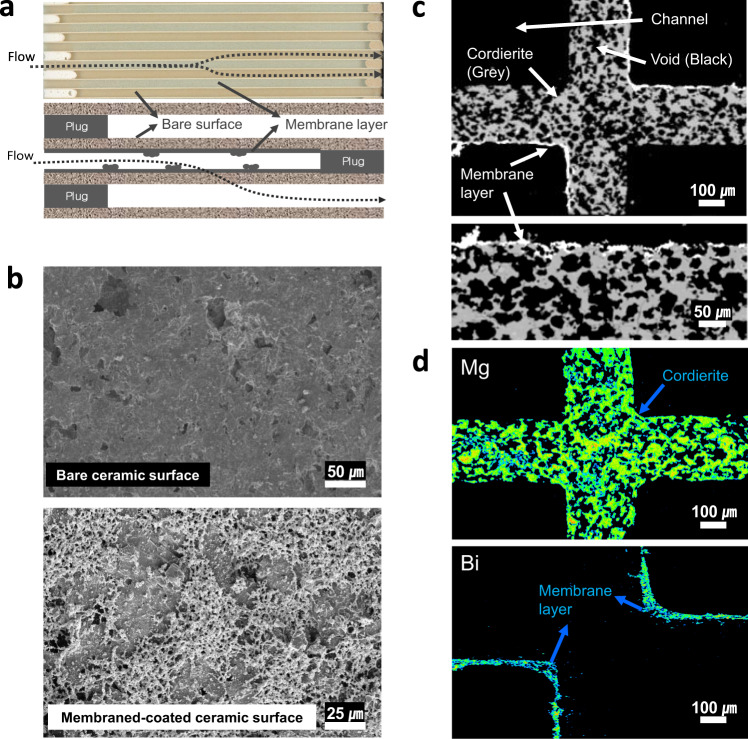
CCF coated by inorganic membranes. **a** Inner channels of CF and illustration of PM removal by wall-flow filtration in the CF. **b** SEM images before and after inorganic coating to fabricate the membrane on the CF cell surfaces. **c** SEM image of cross-sectional sliced CF by moulding and polishing: void of wall (black), membrane layer (white), and wall comprising cordierite (grey). **d** Distribution of main elements of the wall (Mg) and membrane (Bi) by SEM-EPMA element mapping.

Figure 2a illustrates the internal cross-section morphology along the filter length describing a specific feature of the CCF. According to the filtration mechanism of the CF, an air flow with PMs and gaseous air pollutants that entered into the filter would penetrate through the porous wall, where only PMs can be captured on and/or in the pores of the wall. The core of the CCF that removes PM is obtained by an additional surface coating treatment of metal oxide, known as the “membrane”. The morphology of the porous surface with microstructures before and after the membrane coating on the bare-CF can be seen in the scanning electron microscope (SEM) images (Fig. 2b, Supplementary Fig. 3). The membrane with a net-type shape was uniformly spread over the wall surface in the filter channel. The vertical cross-section of the CF shows well-coated membrane layers on the inner wall surface (Fig. 2c), and the characteristic membrane component (Bi) is observed along the wall, which is mainly composed of Mg in cordierite (Fig. 2d). The mean pore size after membrane coating was reduced from 11.6 to 8.2 μm while maintaining the porosity of CF (Supplementary Fig. 3c). It is well known that the pore size reduction could affect permeability related to filter efficiency. In particular, the control of mean (average) pore size plays a critical role in determining the quality factor, including filter efficiency (FE) and pressure drop^22,23^. We evaluated the filter efficiency (FE, %) and pressure drop (Δp, Pa) under a single-pass test condition of an air flow of 1 m/s by using a customised aerodynamic equipment. Compared to the bare-CF, the membrane coated filter achieved an enhanced PM_10_ (particulate matter, size below 10 μm) FE of 98% and PM_2.5_ (particulate matter, size below 2.5 μm) FE of 97.7%, respectively (Table 1). It plays an important role in successfully removing PMs by cake filtration mechanism beyond the initial deep-bed filtration through the CF^7^. Here, we improved the filtration mechanism by membrane coating, which resulted in a significant increase in the FE of PM_1_ (particulate matter, size below 1 μm) as detailed in Table 1.

**Table 1:** Physical properties and measuring data: pressure drop and PM removal efficiency of bare and membraned ceramic filters (at 115 mm)

Ceramic filters		Bare CF		Membraned CF
Material			Cordierite	
Cell density (CPSI) / wall depth (mil)			200/8.0	
Mean pore diameter (μm)		11.6		8.3
Porosity (%)		54.0		55.9
Δp (pressure drop) (Pa) at 1 m/s		62		136
Filter Efficiency (FE) (%) at particle mass	PM_1_	34.9		94.5
	PM_2.5-1_	62.4		99.1
	PM_10-2.5_	83.8		99.6
	PM_2.5_	51.1		97.7
	PM_10_	53.6		98.0

Note that these properties were measured using mercury intrusion porosimetry (MIP).

### VOC removal by coating of synthesized photocatalyst

**Fig. 3:**
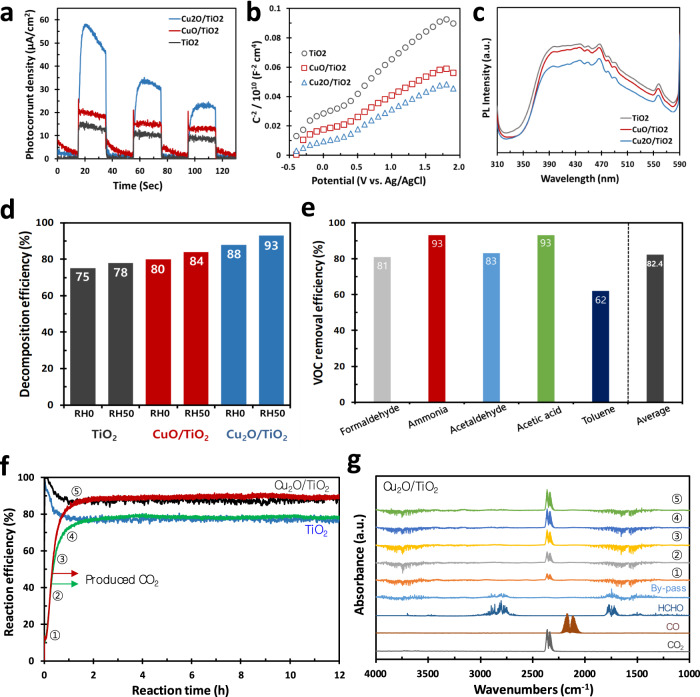
Synthesis of Cu_2_O/TiO_2_ photocatalysts. **a** Photocurrent density by chronoamperometry: using 1 LED with a power density of 50 mW/cm^2^ (365 nm) at 0.5 V (vs. Ag/AgCl). **b** Charge carrier density (cm^−3^) analysis by Mott–Schottky plot: 3.06 × 10^20^ cm^−3^ for Cu_2_O/TiO_2_, 2.81 × 10^20^ cm^−3^ for CuO/TiO_2_, and 1.73 × 10^20^ cm^−3^ for TiO_2_ at flat band potential (E_FB_) of −0.4 V. **c** Photoluminescence: 300-nm excitation by UV and response emission of 310 to 590 nm on the powder samples. **d** VOC (HCHO) decomposition efficiencies of Cu_2_O/TiO_2_, CuO/TiO_2_, and TiO_2_ catalysts. Note that the efficiencies were calculated by ratio of CO_2_ produced by the PCO reaction to a given concentration of HCHO gas. The detailed reaction conditions are available in the supplementary materials. **e** VOC removal efficiencies of five VOCs over the honeycomb (100 CPSI) coated by the Cu_2_O/TiO_2_ catalyst. The 100 L chamber test sequentially proceeded with each VOC at 10 ppm in an air balance for 30 min. This test was conducted three times for each VOC gas. The final average value indicated the arithmetic mean of the efficiencies. **f** VOC reaction efficiencies (HCHO removal and CO_2_ production) of CCF coated with Cu_2_O/TiO_2_ and TiO_2_ catalysts. Detailed reaction conditions are available in the supplementary materials. **g** FT-IR spectra at different stages of the reaction corresponding to the five points shown in Fig. 3f. Note that the FT-IR spectrum of by-pass, and gaseous FT-IR spectra of HCHO, CO, and CO_2_ gases are indicated as per the references.

To remove VOC by the CCF, we developed a Cu_2_O-TiO_2_ catalyst based on a TiO_2_ (anatase) photocatalyst. Cu_2_O having Cu^1+^ can act as a co-catalyst in the conduction band as an electron acceptor, further producing ROS such as superoxide anion (∙O_2_^−^) (Fig. 1)^24,25^. The oxidation state of Cu on the surface of TiO_2_ was confirmed to be Cu^1+^ by X-ray photoelectron spectroscopy (XPS) analysis and Raman spectroscopy (Supplementary Fig. 4a, b). The basic characterisations, such as UV–visible absorbance and electrochemical impedance spectroscopy (EIS), were measured (Supplementary Fig. 4c, d). The PCO reaction activity of Cu_2_O/TiO_2_ catalyst can be improved because Cu_2_O behaves as an electron acceptor, leading to easy separation of hole–electron pairs and reduction of hole–electron recombination^26^. To verify this mechanism, we evaluated the electrochemical characteristics by measuring the photocurrent density, Mott–Schottky plot, and photoluminescence (PL) (Fig. 3a–c). With these enhanced characteristics of the Cu_2_O/TiO_2_ catalyst, we measured the intrinsic formaldehyde decomposition activity of powder catalysts (TiO_2_, CuO/TiO_2_, and Cu_2_O/TiO_2_) under relative humidity (RH) 0% and RH 50% conditions, resulting in 93% @ Cu_2_O/TiO_2_ > 84% @ CuO/TiO_2_ > 78% @ TiO_2_ at RH 50%, which was higher than that at RH 0% owing to the formation of hydroxyl radical (∙OH) by the oxidation reaction with water (Fig. 3d)^12,14^. Furthermore, we assessed the removal efficiencies with five representative VOCs, namely formaldehyde, ammonia, acetaldehyde, acetic acid, and toluene gas, over 1 g of Cu_2_O/TiO_2_ catalyst coated on honeycomb (Fig. 3e). The average removal efficiency was 82.4%, with the highest being 93% for acetic acid and the lowest being 62% for toluene. Therefore, we confirmed the development of a new TiO_2_-based catalyst via Cu_2_O as co-catalyst, which improves the photocatalytic activity through facile charge separation and high charge carrier density. For the Cu_2_O/TiO_2_ catalyst, we coated similar amounts of catalysts for fabricating CCFs (38–40 g of Cu_2_O/TiO_2_ or TiO_2_ catalysts per apparent volume of the filter (L)), where we initially used the CF without the membrane to confirm the catalyst performance. Enhanced reaction efficiencies (REs: VOC removal and CO_2_ production) of nearly 90% using the Cu_2_O/TiO_2_ catalyst were obtained with CO_2_ as the product gas, while those using TiO_2_ were 76% (Fig. 3f). In particular, we extensively monitored the feasible side gas-phased products of PCO reaction, including CO by Fourier transform infrared spectroscopy (FT-IR) along the reaction time, but these were not detected (Fig. 3g). This implies that CO_2_ was the only product resulting from the PCO reaction. Thus, we concluded that all the reacted HCHO gases were perfectly decomposed into CO_2_ gas by the PCO reactions over the Cu_2_O/TiO_2_ catalyst. We used formaldehyde (HCHO), which is a well-known as highly carcinogenic gas^2^, as a representative VOC for the removal test (see Methods). The light intensity test and ray tracing simulation (Zemax) were conducted to optimise the UVA-LED system (Supplementary Figs. 5 and 6). We set up the UV-activated system optimally with a light source using a 2 × 2 UVA LED array with a suitable light intensity of 38.1 (centre) and 40.8 mW/cm^2^ (side), and a maximum light propagation distance of approximately 30 mm inside the CCF.

### Fabrication of CCF

**Fig. 4:**
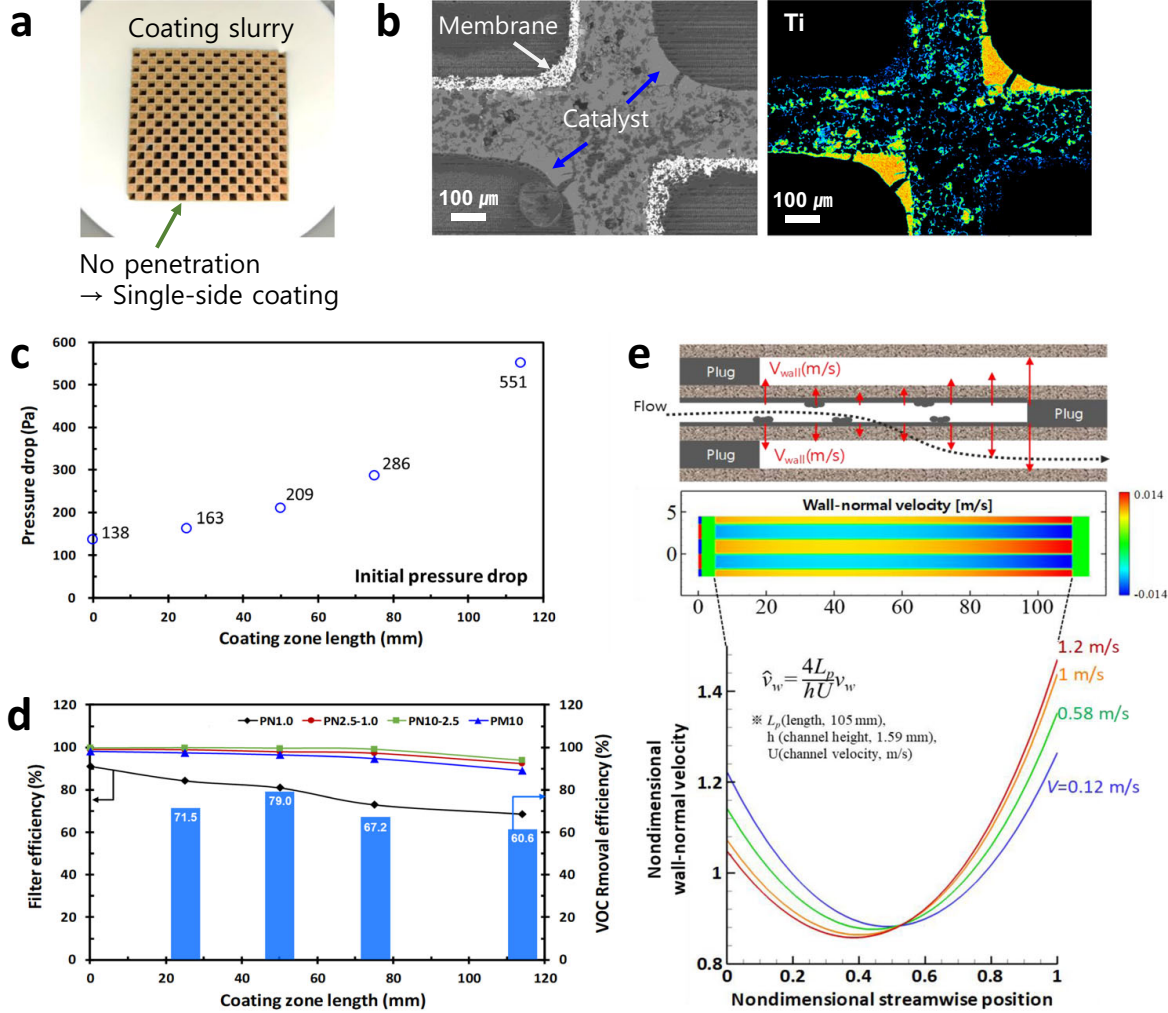
Fabrication of CCF and its simultaneous removal efficiency. **a** Image of a single-side slurry coating with no through-wall penetration. **b** SEM image of CCF fabricated by coating the membrane and catalyst on different surfaces and distributing the main element of the catalyst (Ti) by SEM-EPMA element mapping. **c** Initial pressure drops of CCFs along the catalyst coating zone with lengths of 0 to 115 mm. **d** Filter and VOC removal efficiencies of CCFs along the catalyst coating zone with lengths of 0 to 115 mm. **e** CFD simulation of ceramic filter along the CF length. Wall-normal velocity through the wall under various inlet velocities from 0.12 to 1.2 m/s. Top inset indicates the wall position inside the cell, where the length of the arrow indicated the strength of the wall-normal velocity.

Figure 4a shows a snapshot of the dip-coating of the catalyst with membrane-coated CF for CCF fabrication. When the membrane coated CF was dipped in the catalyst coating slurry, the slurry is not absorbed into the cell, implying no penetration beyond the wall. It is an important design rule that the catalyst should be coated on the outlet channel surface against air flow to prevent deactivation by PM. To observe the inside of the cell (channel), we conducted SEM analysis along with Electron Probe X-ray Microanalysis (EPMA) to confirm the spatial distribution of the main elements in the cell. Figure 4b shows the SEM image of cross-sectional CCF and the element mapping of Ti, which indicates a well-coated catalyst layer on the inner channel surface of CCF. The SEM image shows the wall centre of CCF, which indicates the inside pores (black), coated catalysts (grey), and membranes (white). The mapping image of Ti demonstrates that the catalyst was coated on a single side including the inside wall, implying that no penetration of the coating occurred to the other side (membrane coating zone). Although the inability of the slurry to penetrate the membrane was not investigated in this study, it may be caused by the micro/nano structures generated by the surface treatment (membrane coating) as per Cassie–Baxter^27^. For practical CCF fabrication, we confirmed the relationship among the pressure drop, FE, and RE (or removal efficiency) along the catalyst coating lengths (Fig. 4c, d). The pressure drop increased exponentially along the coating length; however, it was stable up to the 50 mm coating zone. FE decreased gradually as the coating length increased. However, RE achieved an optimum value at the 50 mm coating zone. Thus, we obtained the optimum specifications to fabricate the CCF for practical applications from systematic testing along the catalyst coating length (Fig. 4d, Supplementary Table 2). In addition, to better understand the characterisation along the coating zone length, we investigated the wall-normal velocity along the CF length using CFD simulations (Fig. 4e). The wall-normal velocity passing through the inner wall of the CF increases from above 30% of the total CF length to the maximum at the outlet channel, which implies the photocatalytic reaction might be the most effective within 70% of the total CF length from the outlet.

### Regeneration performances of CCF

**Fig. 5:**
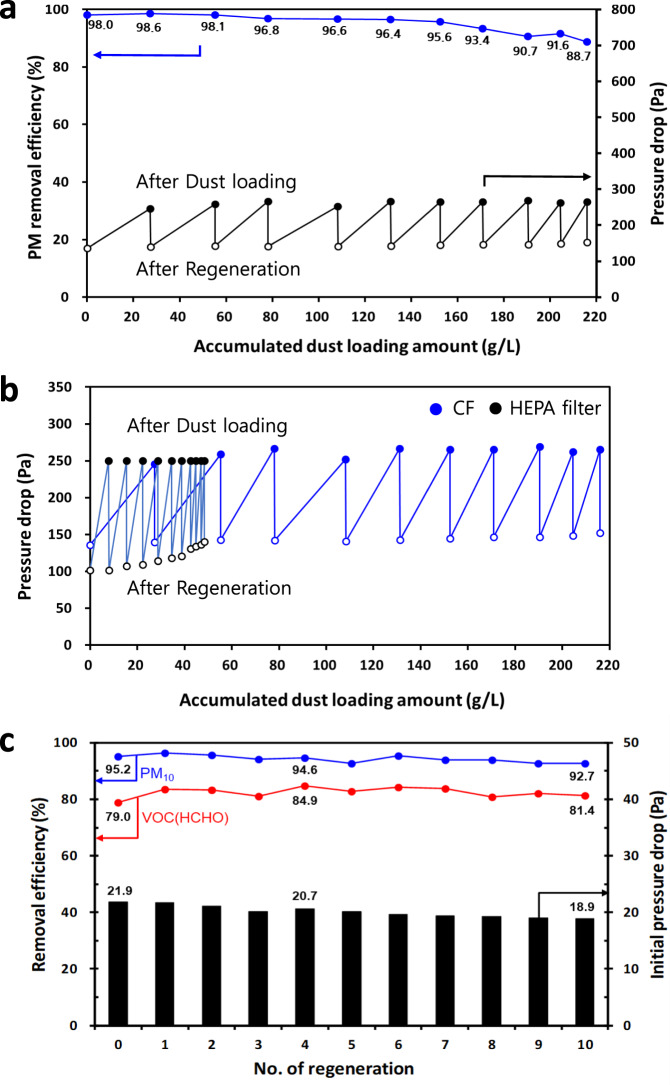
Regeneration performances of CF, HEPA filter, and CCF with water washing. **a** Regeneration performances of PM removal efficiency and pressure drop of CF for ten regenerations by water washing. **b** Dust loading amount profile of the CF (Blue: total dust loading amount, 215.8 g/L, average 21.6 g/L) and the HEPA filter (Black: total dust loading amount, 48.4 g/L, average 4.8 g/L) according to regeneration cycles with water washing. **c** Simultaneous PM FE and VOC RE of CCF for ten regenerations by water washing.

Among the various regeneration methods such as water washing by water flow and sonication, we found that simple water washing in the direction against PM capture in CF or CCF is the most effective way to clean dust and regenerate the initial pressure drop of the filter (Supplementary Fig. 7a, b). The maximum dust loading capacity of CF up to final pressure drop (~250 Pa) was evaluated to be approximately 20–30 g/L, while the normal disposable HEPA or medium filters (MFs) for 6 months have a maximum capacity of 5 g/L (Supplementary Fig. 7c)^28^. For ten regenerations by water washing, the CF achieved an accumulated dust loading capacity exceeding 216 g/L, while maintaining an efficiency loss of barely 9% compared to the high initial efficiency of 98% at a high linear velocity of 1 m/s (Fig. 5a). However, as the regeneration cycle progressed, the commercial HEPA filter rapidly decreased in its dust loading capacity (48.4 g/L for ten regenerations) and could no longer be operated (Fig. 5b). The CF may possibly indicate a usage of 2 years without regeneration exceeding the maximum dust loading capacity four times that of the normal HEPA filters. Thus, it implies that we can use the CF for 20 years through ten regenerations of simple water washing. Finally, we simultaneously achieved PM and HCHO removal using CCF (Fig. 5c). For the CCF with Cu_2_O/TiO_2_ catalyst, the PM_10_ FE, HCHO removal efficiency, and pressure drop initially were about 95%, 82%, and 20 Pa at 10 L/min (linear velocity of 0.12 m/s), respectively. Unprecedentedly, these high performances are maintained even after ten regenerations with simple water washing. This result is firstly demonstrated by the CCF as a new class of filter realised using our main concept suggested in this study. Thus, with simple water washing, we achieved the facile regeneration of the CCF.

### Application for air purification system

**Fig. 6:**
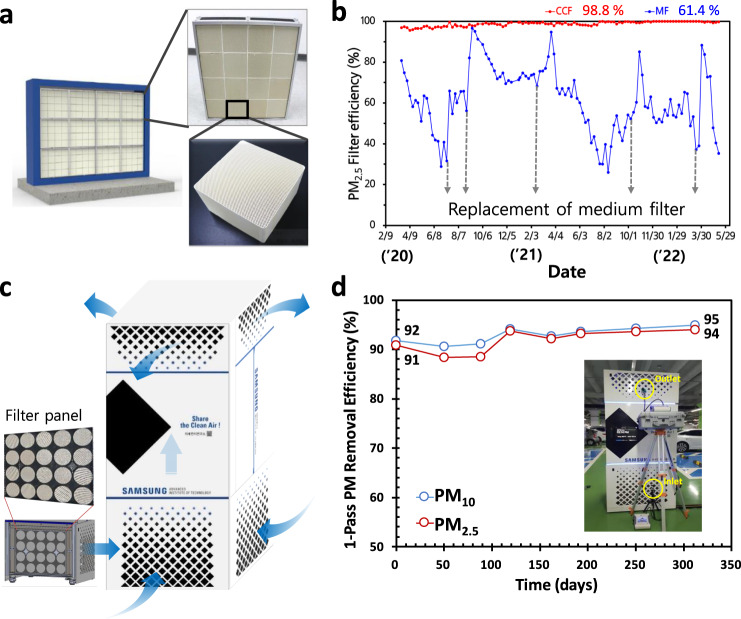
CF applications for sharing the clean air. **a** CF panel system installed at the HVAC facility of the building. **b** PM_2.5_ FE of CF and MF measured for 30 months in the building. **c** Free-standing proto-type CF system. **d** FE of PM_10_ and PM_2.5_ of the proto-type CF system measured for approximately 12 months. The efficiency was measured using a single-pass test method, which assessed the concentration of inlet and outlet PMs using an isokinetic sampling probe, as shown in the inset.

Prior to using the CCF as a prototype for various applications, we first applied the CF panel system to purify the air entering a building to replace current commercial MF (Ultra Low Pressure Drop (ULPD) in heating, ventilation, and air conditioning (HVAC) systems (Fig. 6a). Through this, we confirmed practically that PM_2.5_ FE remains higher than 98% for 30 months without replacement and regeneration of the CF panel system, while the MF showed low FE (62%) and required replacement every 3–6 months (Fig. 6b). In addition, we developed a free-standing air purification system applying CF as another type of proto system to purify the air in the underground parking lot (Fig. 6c). Note that installation of UV light system for CCF performance is still in progress. The FEs of PM_10_ and PM_2.5_ of the system are still observed above 90% for about 12 months for a flow rate of 4000 m^3^/h (Fig. 6d). Note that all the evaluations are in-operation by now. Furthermore, we conducted a CFD simulation to predict the air flow and PM concentrations in huge underground parking lot (volume of 96,000 m^3^ for 600 cars). After 21 CCF proto systems were located, air flow was more uniformly distributed without a quiescent zone, and the overall volume averaged PM_10_ concentration showed a surprising reduction of 32% (Supplementary Fig. 8). To confirm the prediction result of the CFD simulations, we practically test the spatial efficiency using 21 installed CCF proto systems.

## Discussion

In summary, we first designed a new class of facile regenerable CCF for the simultaneous removal of PM and VOC under ambient conditions using membraned porous wall flow filtration and UV-activated photocatalyst in the porous CF for air purification. The structurally innovative design of CCF by two components (membrane and photocatalyst) can offer a great advantage of space efficiency to simultaneously remove various air pollutants. Indeed, the new filter system based on our proposed concept exhibited a PM FE of about 95% and VOC RE of about 82% with perfect PCO reaction to form CO_2_ under single-pass air flow condition. The Cu_2_O/TiO_2_ catalyst can improve the VOC (HCHO) RE by trapping electrons from TiO_2_ as well as by preventing recombination of electron–hole pairs on the catalyst. Furthermore, we demonstrated that the CCF filter can be fully regenerated by simple water washing, and the high removal performance of the filter is sustained even after ten regeneration cycles. The CCF can possibly indicate a usage of 2 years without regeneration, with nearly four times the maximum dust loading capacity than that of conventional HEPA filters, suggesting long-lifetime for 20 years through ten regeneration cycles via simple water washing. The facile regenerable, long-lifetime filter system proposed in this study is expected to provide a new paradigm for indoor and outdoor air purification through sustainable technology.

## Methods

### Preparation of Cu_2_O/TiO_2_ catalyst

The Cu_2_O/TiO_2_ catalyst was prepared by an impregnation method. CuCl_2_ (0.5 wt.%) was dispersed in a TiO_2_ suspension. The suspension was heated at 90 °C and maintained for 1 h in a water bath under stirring. Glucose and NaOH were added to the prepared suspension, which was heated at 90 °C and maintained for 1 h in a water bath under stirring and then dried at 110 °C. Glucose and NaOH were added to reduce CuO to Cu_2_O to control the oxidation state of Cu. The chemicals used in this study included TiO_2_ (ST-01, anatase, Ishihara Inc., Japan), D-(+)-Glucose (Aldrich, USA), CuCl_2_ (Aldrich, USA), and NaOH (Aldrich, USA).

### Catalyst characterisation

The UV-visible spectra of the catalyst were recorded by a UV-visible spectrophotometer equipped with a diffuse reflectance attachment (Solidspec-3700, Shimadzu, Japan). The catalysts were characterised by X-ray photoelectron spectroscopy (XPS), Raman spectroscopy, and Brunauer–Emmett–Teller (BET) surface area analysis. The (photo)electroanalytical measurements were obtained through the photocurrent density, electrochemical impedance spectroscopy (EIS) Nyquist plot, and Mott–Schottky plot. Photoluminescence (PL), for evaluating the charge recombination rate, was also performed. A single compartment cell with a three-electrode configuration was employed, which included working, Pt counter, and an Ag/AgCl reference electrodes under UV irradiation (at 365 nm LED).

### Preparation of CCF: membrane coating

The Commercial CFs were provided by Corning. The membrane coating in the filter was prepared from metal oxide nanoparticles. The nanoparticle deposition in the filter was performed by passing the particle-laden flow through a targeting filter in the gas-phase during the membrane coating process. Finally, the high porous net-type membrane was prepared after calcination. The CF wall surface was measured using an image from a scanning electron microscope (SEM) (FE-SEM JEOL, JSM-7900 and SEM JEOL, SEM-5610). To observe the SEM image of the cross-sectional CF wall, the CCF fragment was moulded in epoxy and polished to reveal the CF wall surface. In addition, SEM analysis equipped with Electron Probe X-ray Microanalysis (EPMA, EPMA-1720, Shimadzu, Japan) was employed to observe the distribution of main elements.

### Preparation of CCF: catalyst washcoating

The CCFs were prepared by washcoating TiO_2_ (ST-01) and Cu_2_O/TiO_2_ (SAIT) catalysts on a 200/8.0 CPSI/mil membrane coated with CFs, conducted by a simple dipping method. Before coating, the catalyst powder was completely suspended in water using a wet-type agitator bead mill (LabStar LS1, NETZSCH, Germany) to maintain a well-mixed solution during washcoating. The mean particle size of the powder in the slurry was 1.84 and 2.57 µm for the TiO_2_ and Cu_2_O/TiO_2_ catalysts, respectively, as determined by the particle size analyser (1090, CILAS, France). The dipping for washcoating was maintained for approximately 2–3 min. The solid content of the catalyst slurry was 40 g/L. Note that g/L indicates the catalyst weight (g) over the apparent volume of CF (L). The wall surface of CCF was measured using an image from a scanning electron microscope (SEM) (FE-SEM, SU-8030, Hitachi, Japan). The catalyst was coated with reasonable uniformity; however, the catalyst thickness on the wall surface could not be quantitatively measured owing to its high porosity which exceeded 50%. The CCFs after washcoating were dried overnight at 80–200 °C.

### Regeneration method by simple water washing

The CCF regeneration was conducted by simple washing using distilled ionised water. After performing the PM and VOC removal evaluations, the CCF was treated by water flow against the PM capture direction for 10 min. After this simple regeneration, the filter was dried overnight at 80–100 °C under vacuum conditions.

### Evaluation of removal efficiencies of PM and VOCs

For PM removal performance evaluation, the single-pass test for CCF was conducted using customised aerodynamic equipment. The pressure drop was also measured during the test. The air flow velocity was maintained at 1 m/s. The dimensions of the prepared CCF sample were 38 × 38 × 115 mm. For PM generation, a 1.5 wt.% KCl solution was used and the dried KCl particles were exposed to the aerodynamic equipment for the single-pass test. Also, Arizona dust (A1 dust, SiO_2_: main component) was mainly employed to compare the regeneration performance between CCF and HEPA. Note that, however, KCl particles had to use to avoid the interference effect such as adsorption of VOC on SiO_2_ (Arizona dust) to conduct the simultaneous removal test of PM and VOC. The particle numbers and weights before and after filtration were obtained by an optical particle sizer (OPS) analyser (Model 3330, TSI, USA). The intrinsic powder catalyst-assisted VOC (HCHO) removal activity was performed in the continuous-flow mode. The VOC gas was continuously fed into the stainless-steel photo-reactor with quartz window at a total flow rate of 0.5 L/min under RH 0% and RH 50% conditions at 25 ^o^C. For the removal test of five VOCs, we used 100 CPSI honeycomb blocks with size of 35 × 70 × 10 mm coated with 1 g of Cu_2_O/TiO_2_ powder. The 10 ppm decrease of each VOC by photocatalytic reaction was continuously measured in a 100 L chamber test for 30 min. The measurements were sequential in the following order: formaldehyde, ammonia, acetaldehyde, acetic acid, and toluene gases. For CCF, the customised Teflon reactor was equipped with an optical polished quartz window at a total flow rate of 10 L/min. The HCHO concentration in the air balance was 25 ppm. The sample in the reactor was illuminated using a 365-nm UV-LED (Fibre Optics Korea Co., Republic of Korea). The concentrations of consumed HCHO and formed CO_2_ were monitored using an FT-IR spectrometer (I4001-E, MIDAC Co., USA) to calculate the VOC removal efficiency and produced CO_2_ gas, respectively. For simultaneous PM and VOC removal test, we fitted the customised aerodynamic equipment with an optical polished quartz window to apply the UV-LED. The test was performed at a total flow rate of 10 L/min where KCl particles as PM and HCHO as VOC were included in the air flow. The HCHO concentration in the air balance was 10 ppm. The pressure drop, PM concentration, and VOC (reactant) and CO_2_ (product) concentrations were measured using pressure gauges installed in the customised aerodynamic equipment, OPS, and FT-IR spectrometer for the same time assessment, respectively.

### CFD and optic simulations

A CFD simulation was conducted using commercial CFD code (ANSYS Fluent 2020R2). The 3D steady Reynolds-averaged Navier-Stokes (RANS) equations, together with the realisable *k-ε* model and passive scalar transport equation with gradient-diffusion hypothesis were solved for the fluid flow and dispersion of PM, respectively, where PM was regarded as a passive scalar and the turbulent Schmidt number was taken as 0.7^29,30^. The traffic-related PM emission and the PM removal process of air purification systems were modelled as a source/sink term in the scalar transport equation (see Supplementary information for details). The SIMPLE algorithm was used for pressure-velocity coupling, and second-order discretisation schemes were applied for all spatial derivatives. Ray tracing simulation was performed using Zemax Optical Design Software. All parameters of the LED source were modelled in accordance with manufacturer specifications. For the simulation, surface characterisation of the TiO_2_ coating was considered to have an absorption/reflection ratio of 73/27 based on the experimental results.

## Supplementary information

Supplementary Information

Peer Review File

## Data Availability

The data that supports the findings of this study are available from the corresponding author on reasonable request.
