# Supplementary material for: Long-lifetime water-washable ceramic catalyst filter for air purification
Source: Nat Commun. 2023 Feb 15;14:520. doi: 10.1038/s41467-023-36050-w (PMC9932083; doi:10.1038/s41467-023-36050-w)
Supplement: Supplementary file 1 — Supplementary Information [file 41467_2023_36050_MOESM1_ESM.pdf]

Supplementary Information

Long-lifetime water-washable ceramic catalyst filter for air purification

Hyuk Jae Kwon[1,†,*], Dong Sik Yang[1,†], Min Seok Koo[1,†], Sang Min Ji[1], Joonseon Jeong[1], Sehyeong Oh[1], Su Keun Kuk[1], Hyeon-su Heo[1], Dong Jin Ham[1], Mijong Kim[1], Hyoungwoo Choi[1], Jong-Min Lee[1], Joong-Won Shur[2], Woo-Jin Lee[2], Chang-Ook Bin[2], Nikolay Timofeev[3], Huiqing Wu [4], Liming Wang[4], Taewoo Lee[5], Daniel J. Jacob[6], Hyun Chul Lee[1]*

[1]Air Science Research Center (ASRC), Samsung Advanced Institute of Technology (SAIT), Samsung Electronics Co., Ltd.; 130 Samsung-ro, Yeongtong-gu, Suwon-si, Gyeonggi-do 16678, Republic of Korea.

[2]Corning Technology Center Korea, Corning Precision Material Co., Ltd.; 212 Tangjeong-ro, Tangjeong-myeun, Asan-si, Chungcheongnam-do 31454, Republic of Korea.

[3]Corning Scientific Center; 26, lit.A, Shatelena St., St. Petersburg, 194021, Russia.

[4]Corning Research Center China; Block H, 200 Jinsu Road, Shanghai 201206, China.

[5]Heesung Catalysts Co.; #507-1Da, 91, Somanggongwon-ro, Siheung-si, Gyeonggi-do, 15088, Republic of Korea.

[6]John A. Paulson School of Engineering and Applied Sciences; Harvard University, Cambridge, MA 02138, USA.

†These authors contributed equally: Hyuk Jae Kwon, Dong Sik Yang, Min Seok Koo

*Corresponding authors. Email: hj13.kwon@samsung.com, hc001.lee@samsung.com

This PDF file includes:

Supplementary Figures 1 to 8

Supplementary Tables 1 to 2

Supplementary Note 1: Pressure drop prediction for CF selection

Supplementary Note 2: Ray tracing simulation

Supplementary Note 3: CFD simulation for underground parking lot

Supplementary References

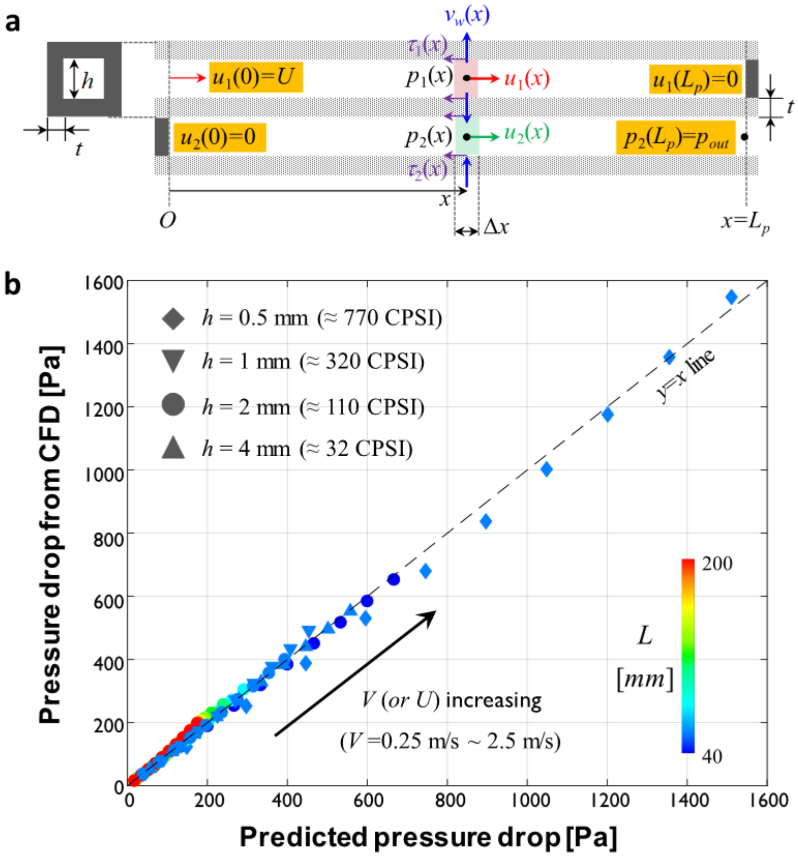

Supplementary Fig. 1 Modelling and CFD simulation of the CF. a Modelling of the CF, where u: streamwise velocity, v_w: transverse velocity at the porous wall, p: pressure, and τ: wall shear stress. **b** Comparison between the pressure drop from CFD and model-predicted pressure drop of CF.

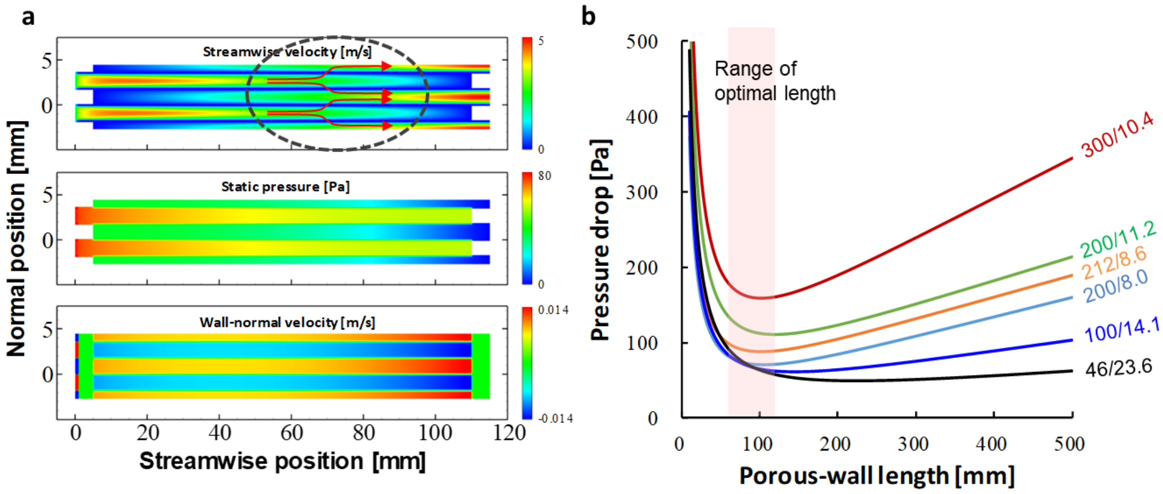

Supplementary Fig. 2 Modelling and CFD simulation of the ceramic filter. a Linear velocity and static pressure inside the CF cells as per CFD simulation. **b** Model prediction of the pressure drop with respect to the porous-wall length of various CF CPSIs to ascertain the range of optimal length.

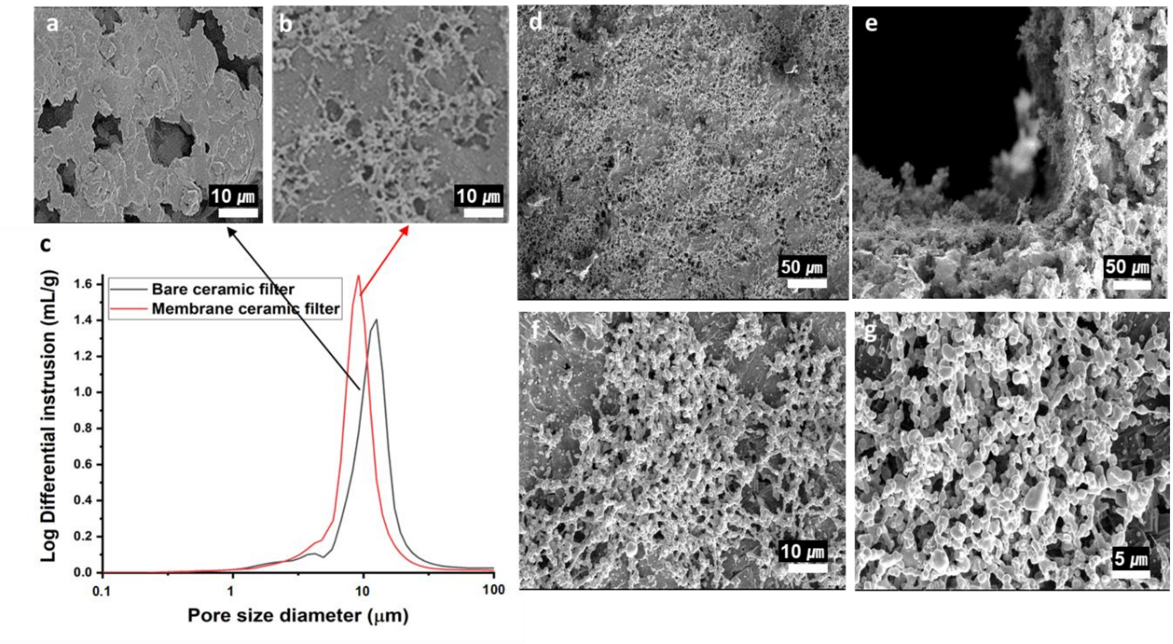

Supplementary Fig. 3 Membrane coating on the ceramic filter: SEM images of CCF wall surfaces. a Bare ceramic filter. **b** CF after membrane coating. **c**, Distribution of CCF pore size diameter before and after membrane coating. **d–g** Various magnifications of SEM images of membrane-coated CCF: Surface image (× 1,000) (**d**), Cross-sectional image (× 1,000) (**e**), Surface image (× 5,000) (**f**), and Surface image (× 10,000) (**g**).

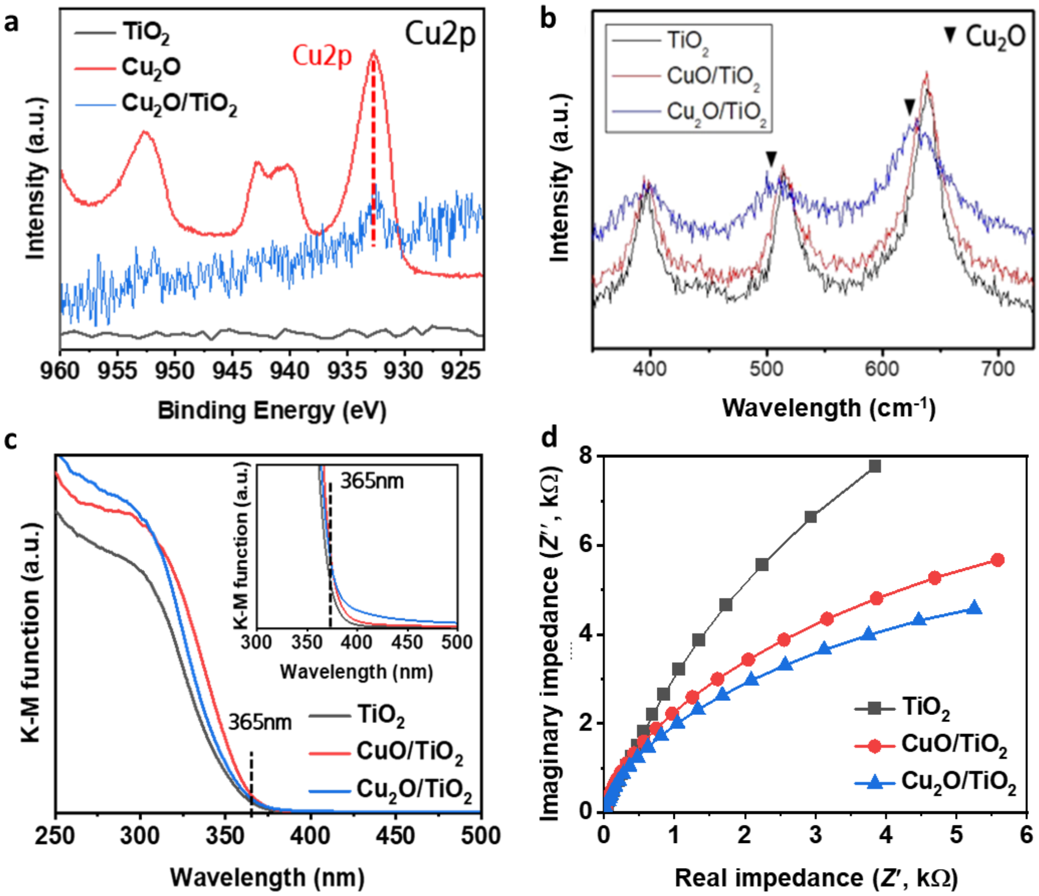

Supplementary Fig. 4 Catalyst characterisations of CuO/TiO_2, Cu_2O/TiO_2, Cu_2O, and TiO_2 catalysts. **a** XPS spectra assigned to the Cu2p position. **b** Raman spectra. **c** UV-visible absorbance. **d** Electrochemical impedance spectroscopy (EIS) Nyquist plot.

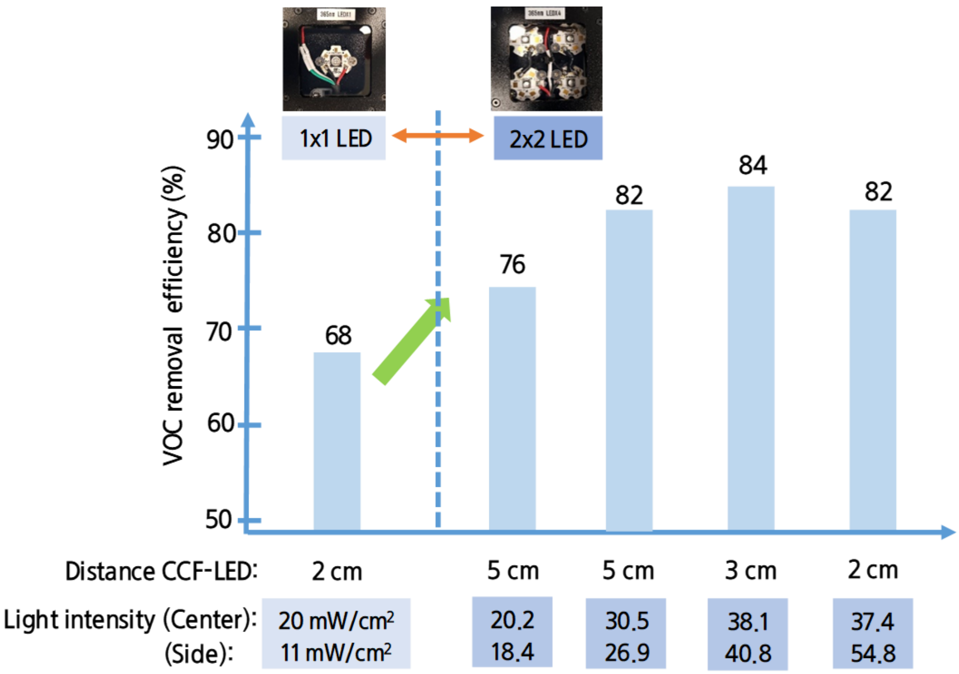

Supplementary Fig. 5 VOC removal efficiency of CCF along with distance of CCF-LED and light intensity of 1-LED and 2×2 LED array. The reaction test used LEDs (1 LED and 2×2 LED array) with a 365-nm wavelength. The reaction on the Cu_2O/TiO_2 catalyst was performed under HCHO at 25 ppm in an air balance at RT and RH 50% with a flow rate of 10 L/min. The catalyst coating was 43.4 g/L.

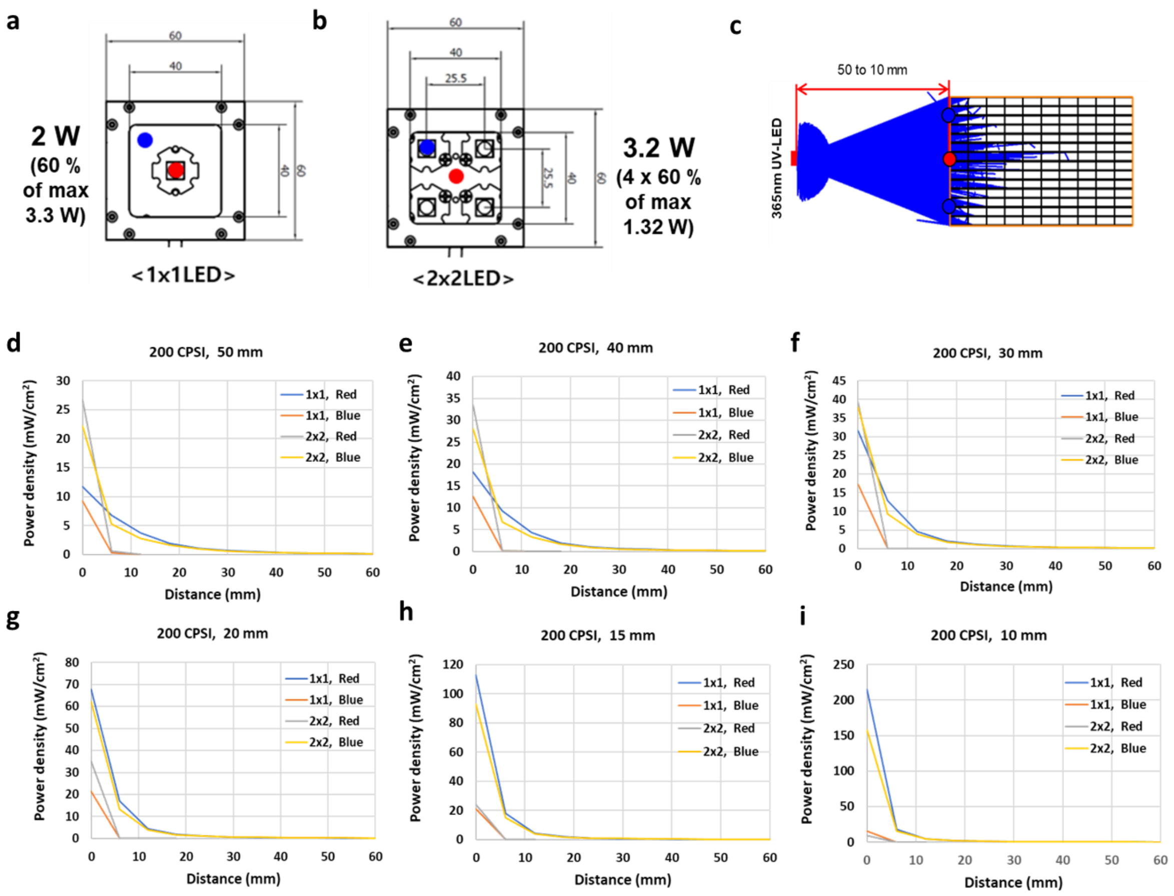

Supplementary Fig. 6 The light intensity and light propagation distance with respect to the CCF–LED distance using ray tracing simulations for 1-LED and 2 × 2 LED arrays. **a, b** Illustration of 1-LED (**a**) and 2 × 2 LED arrays (**b**). **c** Ray tracing result (Top view): Rays (Blue). **d–i** Power distance with respect to the distance between the light source and CCF: 50 (**d**), 40 (**e**), 30 (**f**), 20 (**g**), 15 (**h**), and 10 mm (**i**).

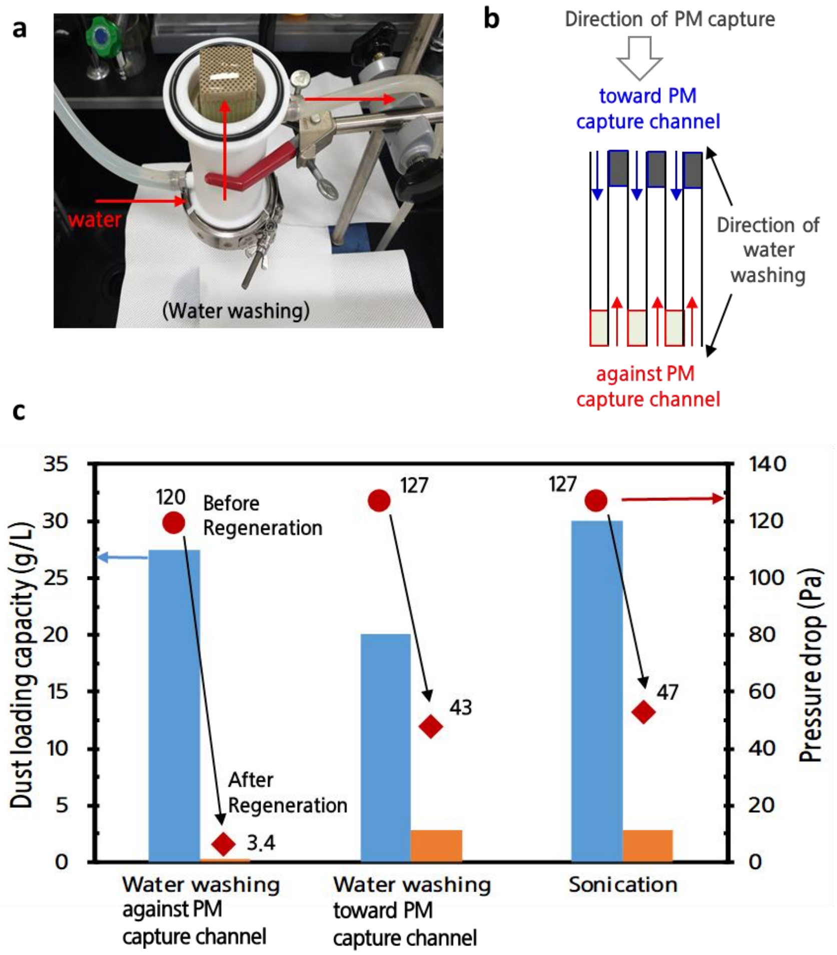

Supplementary Fig. 7 Regeneration of CCF. a, b Image and illustration of facile water washing of CCF. **c** Regeneration performances (dust loading capacity and pressure drop) with respect to water washing and sonication.

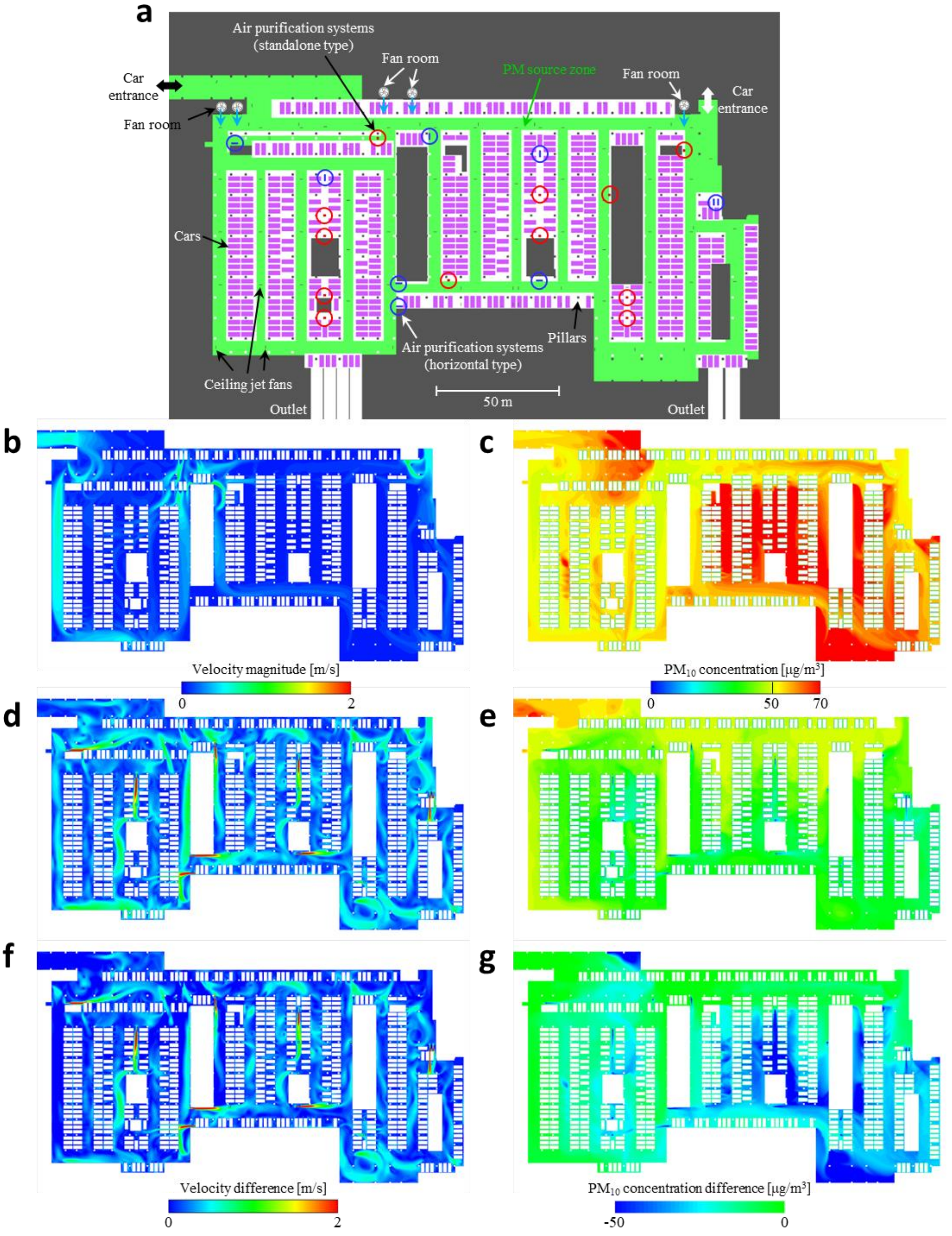

Supplementary Fig. 8 Results predicted from CFD simulation for an underground parking lot. **a** Computational domain (top view). **b** Contours of air velocity magnitude with only the fan rooms operating. **c** Contours of PM_{10} concentration with only the fan rooms operating. **d** Contours of air velocity magnitude with the installation of 21 CCF proto systems

and ceiling jet fans. **e** Contours of PM_{10} concentration with the installation of 21 CCF proto systems and ceiling jet fans. **f** Contours of the subtracted velocity magnitude (for a difference). **g** Contours of the subtracted PM_{10} concentration. The locations of the CCF proto systems are represented by red (standalone, 4,000 m^3/h) and blue (horizontal, 8,000 m^3/h) circles in **a**. All results were obtained at 1 m above the floor level in **b–e**.

Supplementary Table 1 Physical properties of various commercial filters, and wall length and pressured drop estimated from the pressure drop model.

Ceramic cell density (CPSI/mil)	200/11.2	200/8.0	212/8.6
Channel height (mm)	1.51	1.59	1.53
Wall thickness (mm)	0.284	0.203	0.218
Nominal porosity (%)	50.7	55.8	55.3
Median pore diameter (μm)	15.03	13.2	13.5
Estimated permeability (m^2)	9.37E−13	1.01E−12	1.01E−12
Optimal porous-wall length (mm)	119	105	102
Estimated pressure drop (Pa)	111	71	89

Supplementary Table 2 CCF performance along the catalyst coating zone with lengths of 0 to 115 mm.

CCF samples		0 mm coating	25 mm coating	50 mm coating	75 mm coating	115 mm coating
Catalyst (g)		0	1.89 (52.2 g/L)	3.02 (41.7 g/L)	4.58 (42.2 g/L)	6.41 (38.8 g/L)
VOC removal efficiency (%) at 10 L/min		-	71.5	79.0	67.2	60.6
Initial Pressure drop (Pa) at 1 m/s (86.8 L/min)		137.6	163.2	208.9	286.2	551.0
Initial Filter Efficiency (%) at particle mass	PM_1	95.3	92.2	89.8	85.5	80.8
	$PM_{2.5–1}$	99.1	99.1	98.3	97.8	92.9
	$PM_{10–2.5}$	99.5	99.8	99.5	99.1	93.2
	$PM_{2.5}$	97.9	97.0	95.8	93.9	88.6
	PM_{10}	98.1	97.4	96.4	94.7	89.1

Supplementary Note 1: Pressure drop prediction for CF selection

The model predicting the pressure drop of the CFs was investigated using continuity and momentum equations and Darcy's law for porous wall developed by Masoudi et al.[1] and Konstandopoulos & Johnson[2]. The model accuracy of 32 to 770 CPSI was compared to that of computational fluid dynamics (CFD) simulations (Supplementary Fig. 1). To better understand the flow characteristics inside the CF channels, we investigated the velocity and pressure distributions of the CFs using CFD simulations. Supplementary Fig. 2a shows the contours of the streamwise velocity, static pressure, and wall-normal velocity. Along the streamwise direction, the streamwise velocity decreased at the inlet channel but increased at the outlet channel owing to the through-wall flow. The static pressure decreased along the streamwise direction owing to the skin friction force. The wall-normal velocity was fastest at the end of inlet channel, implying that the pressure drop across the wall was greatest at this position because of Darcy's law. Note that the contours of streamwise velocity and static pressure are at the channel centre plane, whereas the contours of wall-normal velocity are at the wall centre plane. Supplementary Fig. 2b shows the predicted pressure drop of the CFs with the porous-wall length (excluding the plug length). As the porous-wall length decreases to zero, the pressure drop increases because the through-wall velocity is increased. When the porous-wall length approaches infinity, the pressure drop also increases because the viscous friction at the channel wall becomes significant. The optimal porous-wall lengths were observed in the range of 60–120 mm, which is the minimum pressure drop range of 46–300 CPSI (number of cells/wall thickness). Therefore, we selected a suitable commercial CF with approximately 200 CPSI because CPSI values lower than 200 CPSI, such as 46 and 100 CPSI, exhibit low amounts of catalyst per given area for light illumination, whereas CPSI values higher than 200 exhibit a high pressure drop in the optimal range of the filter length.

Supplementary Note 2: Ray tracing simulation

Two cases of filter illumination were considered, as shown in Supplementary Fig. 6: (a) one LED on the central axis and (b) 2x2 LED array. The light source to CCF distance was varied in the range of 10–50 mm. There was a set of detectors inside the CCF at different distances from the front face of the CCF with a step of 5 mm. One set of detectors indicated by a red dot in Supplementary Fig. 6a, b was disposed on the axis of one LED (a) and another set of detectors indicated by a blue dot was in front of one of the 2 × 2 LEDs (b). Supplementary Fig. 6c qualitatively illustrates the depth of light penetration inside the CCF in case of one LED. The longest penetration length was observed to exhibit light rays close to the optical axis and propagate nearly parallel to the cell walls. The power of the slanted light rays decays quickly in the beginning of the cells. The reason for this behaviour is the relatively high absorption/reflection ratio (73/27) characterising the TiO_2 coating used in the modelling. Slanted light rays "attack" the cell walls in the beginning of CCF and lose their power very quickly after a few bounces. Supplementary Fig. 6d–i illustrates the light power density inside CCF at different distances from the front face for the "red" and "blue" detectors and for two light source cases (a and b). The maximum length of light propagation inside the CCF was observed to be 20–30 mm along the CCF length.

Supplementary Note 3: CFD simulation for underground parking lot

For the application of air purification systems equipped with CCFs, we focused on an underground parking lot located in Hwaseong city, Republic of Korea. There are 2 car entrances and 3 fan rooms at the upper side of parking lot (Supplementary Fig. 8a). Two outlets exist at the lower side of parking lot for ventilation. In addition, 84 jet fans were installed in the ceiling aid deliver air. For the CFD simulations, we developed a 3D CAD model of an underground parking lot, including 21 air purification systems (9 horizontal-type for 8,000

m^3/h and 12 standalone-type for 4,000 m^3/h), underground offices, pillars, cars, and ceiling jet fans. In the fan rooms, we applied velocity-inlet boundary conditions which correspond to volumetric flow rates of 71,000, 106,500, and 35,000 m^3/h. In the car entrances and outlets, zero-gauge pressure was assumed. For the other walls, we used a no-slip boundary condition with standard wall functions. The flow rates of the air purification systems were 8,000 and 4,000 m^3/h for the horizontal and standalone types, respectively. In addition, the flow rate of a ceiling jet fan was set at 2,280 m^3/h. For PM, the PM_{10} concentration of intake air flow through the fan rooms was set at 50 μg/m^3. The PM removal process of the air purification systems was modelled as a sink term in the scalar transport equation. The sink term was calculated by the PM removal rate divided by the volume of air purification system, where PM removal rate was determined by the PM removal efficiency and the mass flow rate of PM at the inlet of air purification system. The PM removal efficiency of the air purification system was set at 0.9 based on the experimental results. For the estimation of the traffic-related PM emission, we assumed that the parking lot traffic type to be stagnant city traffic and all vehicles to be passenger cars, i.e. the PM emission per driven kilometre was 0.039 g/km[(3)]. We assumed that the number of vehicles passing the through roads was 1500 cars/day and the mean driven kilometres by each car was 0.5 km/car, from which the daily PM emission of 29.25 g/day was calculated. This PM emission was modelled as a source term, which was uniformly imposed in a volume (represented by a green surface in Supplementary Fig. 8a) with the height in the range of 0–1.5 m[(4)]. Supplementary Fig. 8b shows the contours of velocity magnitude 1 m above the floor level when only the fan rooms are in operation (uncontrolled case). We found some quiescent zones in the middle and lower- parts where the magnitude of velocity was small. Consequently, the PM_{10} concentration was relatively high in those regions because the PM had accumulated (Supplementary Fig. 8c). Supplementary Fig. 8d represents the contours of magnitude of velocity in the presence of air purification systems in conjunction with ceiling jet

fans (controlled case). Well-distributed flow patterns were displayed by air purification systems and ceiling jet fans. As a result, the overall PM_{10} concentration was significantly lower than that of the uncontrolled case, indicating a reduction of 32% by volume-average concentration (Supplementary Fig. 8e). To clearly visualize and isolate the effects of the air purification systems and ceiling jet fans on the flow and PM_{10} concentration fields, we subtracted the flow and PM_{10} concentration fields of the uncontrolled case from those of the controlled case. Supplementary Fig. 8f shows the contours of the subtracted velocity magnitude. The results clearly indicate that the air purification systems and the ceiling jet fans significantly altered the flow patterns of the parking lot. Also, the PM_{10} concentration in quiescent zones is greatly reduced by the air purification systems and the ceiling jet fans (Supplementary Fig. 8g). The results show that the air purification systems with CCFs may be applied for the reduction of PM concentration of semi-indoor spaces, such as underground parking lots, with the ingress of external air.

Note that there are the limitations of the present CFD simulations. We treated the particles as a passive scalar (Eulerian approach), although relatively large particles were considered. Also, we neglected the gravitational settling and deposition of particles, as done in previous studies[3,4]. Therefore, a further study for the comparison of the present results with those from Lagrangian approach and experimental ones (on-site measurements as on-going work) is required to validate the present CFD simulations as a future work.

Supplementary References

1. Masoudi, M. et al. Validation of a model and development of a simulator for prediction the pressure drop of diesel particulate filters. *SAE paper* No. 2001−01−0911 (2001).
2. Konstandopoulos, A. G. & Johnson, J. H. Wall-flow diesel particulate filters-their pressure drop and collection efficiency. *SAE Transactions* **98**, 625−647 (1989).

3. Blocken, B., Vervoort, R. & van Hooff, T. Reduction of outdoor particulate matter concentrations by local removal in semi-enclosed parking garages: a preliminary case study for Eindhoven city center. *J. Wind Eng. Ind. Aerodyn.* **159**, 80–98 (2016).
4. Vervoort, R., Blocken, B. & van Hooff, T. Reduction of particulate matter concentrations by local removal in a building courtyard: Case study for the Delhi American Embassy School. *Sci. Total Environ.* **686**, 657–680 (2019).
